# Supplementary material for: Full-length human GLP-1 receptor structure without orthosteric ligands
Source: Nat Commun. 2020 Mar 9;11:1272. doi: 10.1038/s41467-020-14934-5 (PMC7062719; doi:10.1038/s41467-020-14934-5)
Supplement: Supplementary file 1 — Supplementary Information [file 41467_2020_14934_MOESM1_ESM.pdf]

Supplementary information

Full length human GLP1 receptor structure without orthosteric ligands

Wu et al.

Contents:

Supplementary Tables 1-2
Supplementary Figures 1-6
Supplementary Movies 1-3

Supplementary Table 1: Data collection and refinement statistics (molecular replacement)

	GLP-1R–PF-06372222–Fab7F38[#]
**Data collection**	
Space group	P 2_1 2_1 2_1
Cell dimensions	
a, *b*, *c* (Å)	63.0, 64.7, 322.4
α, β, γ (°)	90.0, 90.0, 90.0
Resolution (Å)	45.7 - 3.20 (3.37- 3.20)*
R_{sym} or R_{merge}	11.6 (62.6)
CC(1/2) (%)	99.8 (52.6)
I / σI	6.5 (1.8)
Completeness (%)	97.8 (96.7)
Redundancy	4.8 (3.9)
**Refinement**	
Resolution (Å)	30.00 - 3.20
No. reflections	21990
R_{work} / R_{free}	0.213 / 0.262
No. atoms	
Protein	6486
Ligand/ion	37 / 1
B-factors (Å^2)	
Wilson	101.7
Protein	136.7
Ligand/ion	154.3 / 175.2
R.m.s. deviations	
Bond lengths (Å)	0.010
Bond angles (°)	0.94

[#]Number of crystals: 21

*Values in parentheses are for highest-resolution shell.

Supplementary Table 2: Primers used in the studies

Primers of 11 mutations

S193C/I196F-F	5'AGTTTCATCCTCCGCGCGTTGTGTGTGTTCTTTAAGGACGCTGCCCTGAAATGGATG
S193C/I196F-R	3'CATCCATTTCAGGGCAGCGTCCTTAAAGAACACACACAACGCGCGGAGGATGAAACT
S225A/M233C-F	5'CTCTTGAGCTACCAAGATAGTCTCGCGTGCCGTTTGGTTTTCCTGCTCTGTCAGTACTGTGTGGCTGCCAACT
S225A/M233C-R	3'AGTTGGCAGCCACACAGTACTGACAGAGCAGGAAAACCAAACGGCACGCGAGACTATCTTGGTAGCTCAAGAG
S271A-F	5'ACAATGGATCTTCCGCCTCTACGTGGCCATTGGTTGGGGCGTCCCCCTGCTCTT
S271A-R	3'AAGAGCAGGGGGACGCCCCAACCAATGGCCACGTAGAGGCGGAAGATCCATTGT
I317C/G318I-F	5'CGTCTGCCCATCCTCTTCGCCTGTATCGTGAACTTCTTGATCTTCGT
I317C/G318I-R	3'ACGAAGATCAAGAAGTTCACGATACAGGCGAAGAGGATGGGCAGACG
K346A/C347F-F	5'AACCTCATGTGCAAGACAGACATCGCATTTAGATTGGCTAAGTCCACCTTGACT
K346A/C347F-R	3'AGTCAAGGTGGACTTAGCCAATCTAAATGCGATGTCTGTCTTGCACATGAGGTT
G361C-F	5'TGACTCTGATCCCATTGCTGTGTACGCACGAGGTTATTTTCGCT
G361C-R	3'AGCGAAAATAACCTCGTGCGTACACAGCAATGGGATCAGAGTCA
E387D-F	5'TCAGATTCATCAAATTGTTCACCGACCTGAGTTTCACTTCGTTCCAGGGA
E387D-R	3'TCCCTGGAACGAAGTGAAACTCAGGTCGGTGAACAATTTGATGAATCTGA

Primers of fusion partner insertion

Fusion partner Rubredoxin-F	5'CCTGTACACGCTCTTGGCTTTCATGAAGAAGTATACCTGT
Fusion partner Rubredoxin-R	3'AGGCGGAAGATCCATTGTTCCTCCACCTCTTCAAATTGGTCC

Primers of GLP-1R mutants in cAMP assay

E127C-F	5'TGGCGTGATTTGTCTGAGTGCTGCGAATCAAAACGCGGTGAACGTAG
E127C-R	3'CTACGTTCACCGCGTTTTGATTCGCAGCACTCAGACAAATCACGCCA
Q211C-F	5'GATGTACTCCACCGCAGCGCAGTGCCATCAGTGGGACGGACTCTTGAG
Q211C-R	3'CTCAAGAGTCCGTCCCACTGATGGCACTGCGCTGCGGTGGAGTACATC
Q37C-F	5'CTCAAGAGTCCGTCCCACTGATGGCACTGCGCTGCGGTGGAGTACATC
Q37C-R	3'CTGACGGCGGTATTCCCTCCACTTGCAAACGGTCTCCCACAGAGAGACA
L379C-F	5'TGGATGAACATGCCAGGGGAACCTGCAGATTCATCAAATTGTTCACCGAGCTG
L379C-R	3'CAGCTCGGTGAACAATTTGATGAATCTGCAGGTTCCCCTGGCATGTTCATCCA

Supplementary Figures:

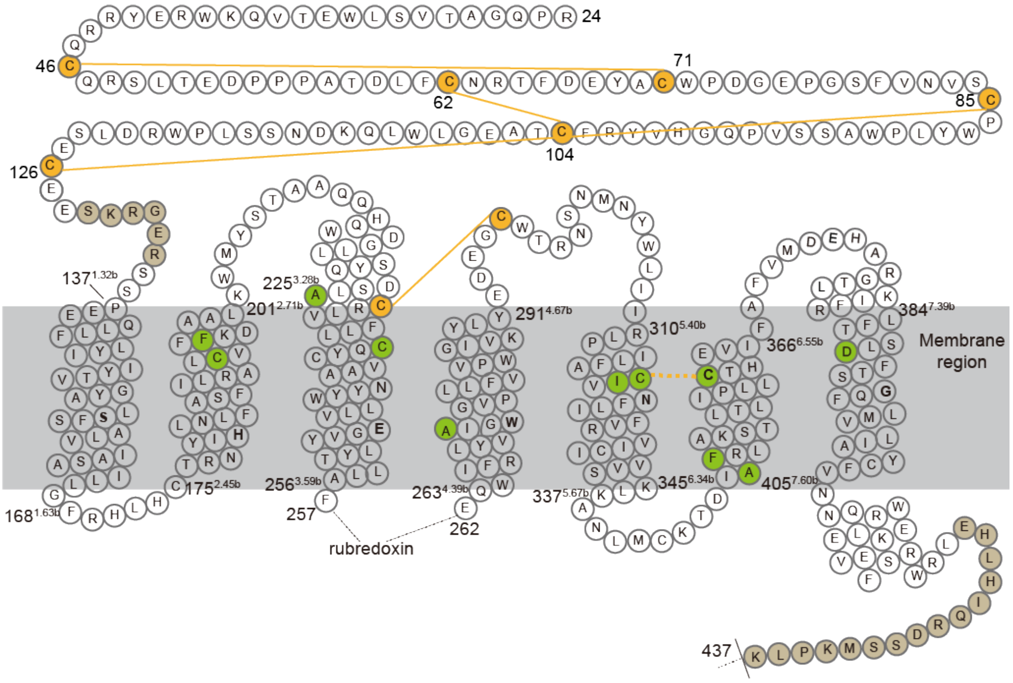

Supplementary Fig. 1. Snake plot of the GLP-R construct used for crystallization. Snake plot of the GLP-1R–rubredoxin fusion construct used for crystallization. Thermo-stabilizing mutations (green) are S193$^{2.63b}$C, I196$^{2.66b}$F, S225$^{3.28b}$A, M233$^{3.36b}$C, S271$^{4.47b}$A, I317$^{5.47b}$C, G318$^{5.48b}$I, K346$^{6.35b}$A, C347$^{6.36b}$F, G361$^{6.50b}$C, and E387$^{7.42b}$D. The most conserved residue among class B GPCRs in each transmembrane helix are indicated in bold. Disordered residues in the structure are shown with brown background. The endogenous and engineered disulfide bonds are shown with solid and dashed orange lines, respectively.

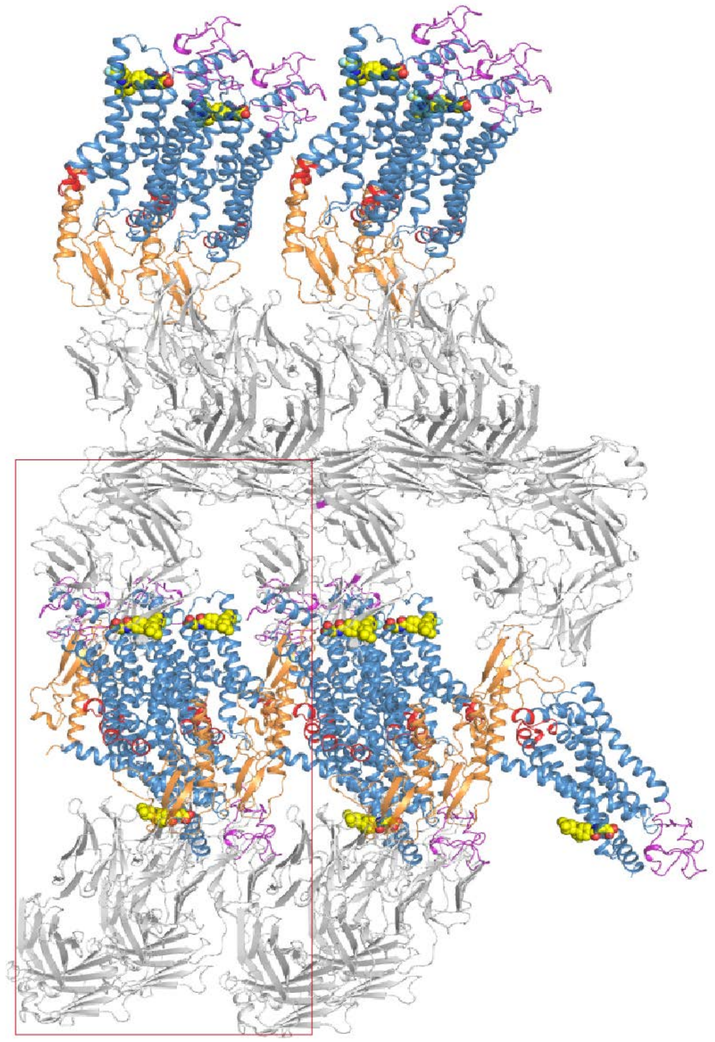

Supplementary Fig. 2. Crystal packing of the GLP-1R–PF-06372222–Fab7F38 complex structure. Fab7F38 is shown in cartoon representation (grey) with the receptor's ECD (orange), ECL1/3 (red) and TMD (blue). The fusion protein (rubredoxin) is in purple and PF-06372222 is displayed as spheres with yellow carbons. The unit cell is indicated by the red box.

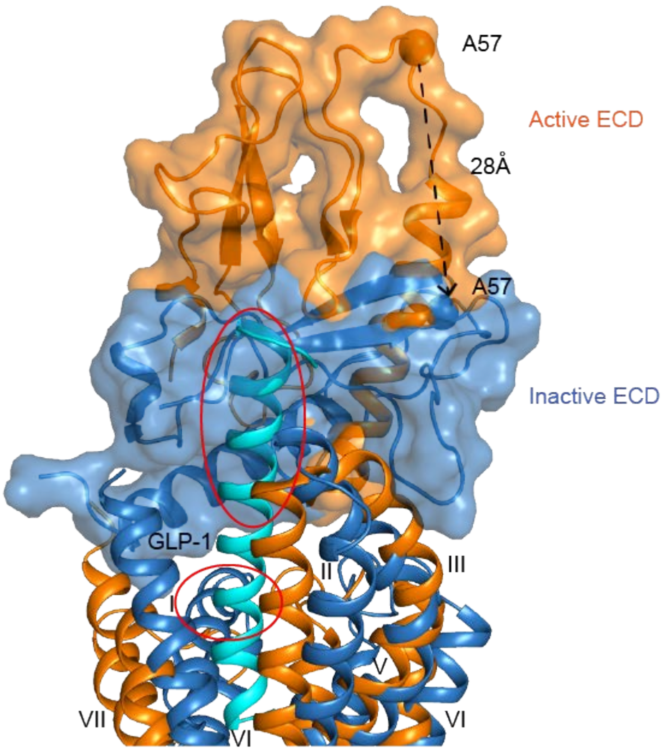

Supplementary Fig. 3. Comparison between inactive GLP-1R crystal structure and active GLP-1R structure. The inactive GLP-1R–Fab7F38 and active GLP-1R–GLP-1 complex structures are superimposed based on TMD alignment. The full-length inactive GLP-1R and active GLP-1R structures are shown as blue and orange, respectively. The ECDs are shown as surfaces and coloured as labelled. The GLP-1 in the active GLP-1R structure is shown in cyan. Red circles indicate where clashes occur when GLP-1 is docked into the inactive structure. The ECD path from the active to inactive conformation is highlighted with a black arrow showing movement of the landmark residue A57.

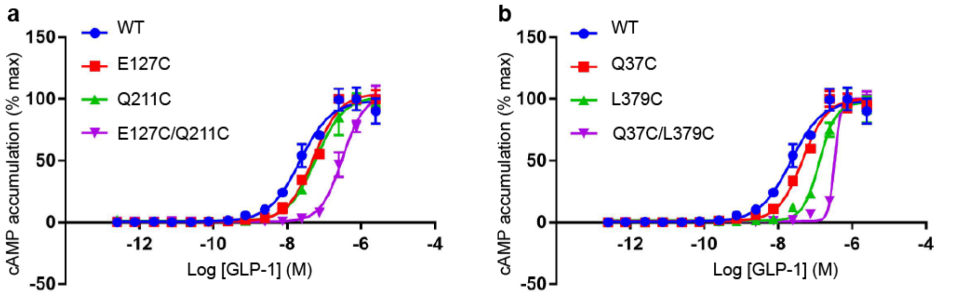

	WT	E127C	Q211C	E127C/Q211C
pEC50(M)	8.44±0.70	8.13±0.86	8.01±0.80	6.09±0.55

	WT	Q37C	L379C	Q37C/L379C
pEC50(M)	8.44±0.70	8.16±0.77	7.54±0.64	6.98 ±0.42

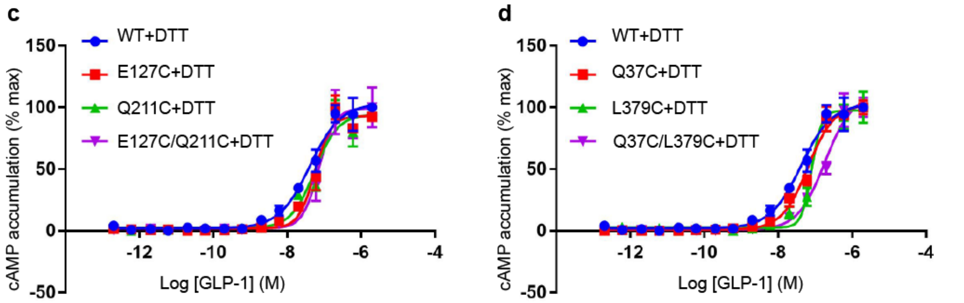

DTT	WT	E127C	Q211C	E127C/Q211C
pEC50(M)	8.23±0.68	8.14±0.89	7.79 ±0.53	7.62±0.45

DTT	WT	Q37C	L379C	Q37C/L379C
pEC50(M)	8.23±0.68	7.98±0.92	7.09 ±0.92	7.96±0.92

Supplementary Fig. 4. GLP-1–induced cAMP accumulation assays. GLP-1–induced cAMP accumulation measurements of wild-type, single point (E127C, Q211C, Q37C, L379C), and double point (E127/Q211C, Q37/L379C) mutants of GLP-1R in the absence (**a**, **b**) or presence (**c**, **d**) of 1 mM DTT. Dose–response curves of were generated from three independent experiments performed in duplicate. Data are shown as means ± s.e.m. Source data are provided as a Source data file.

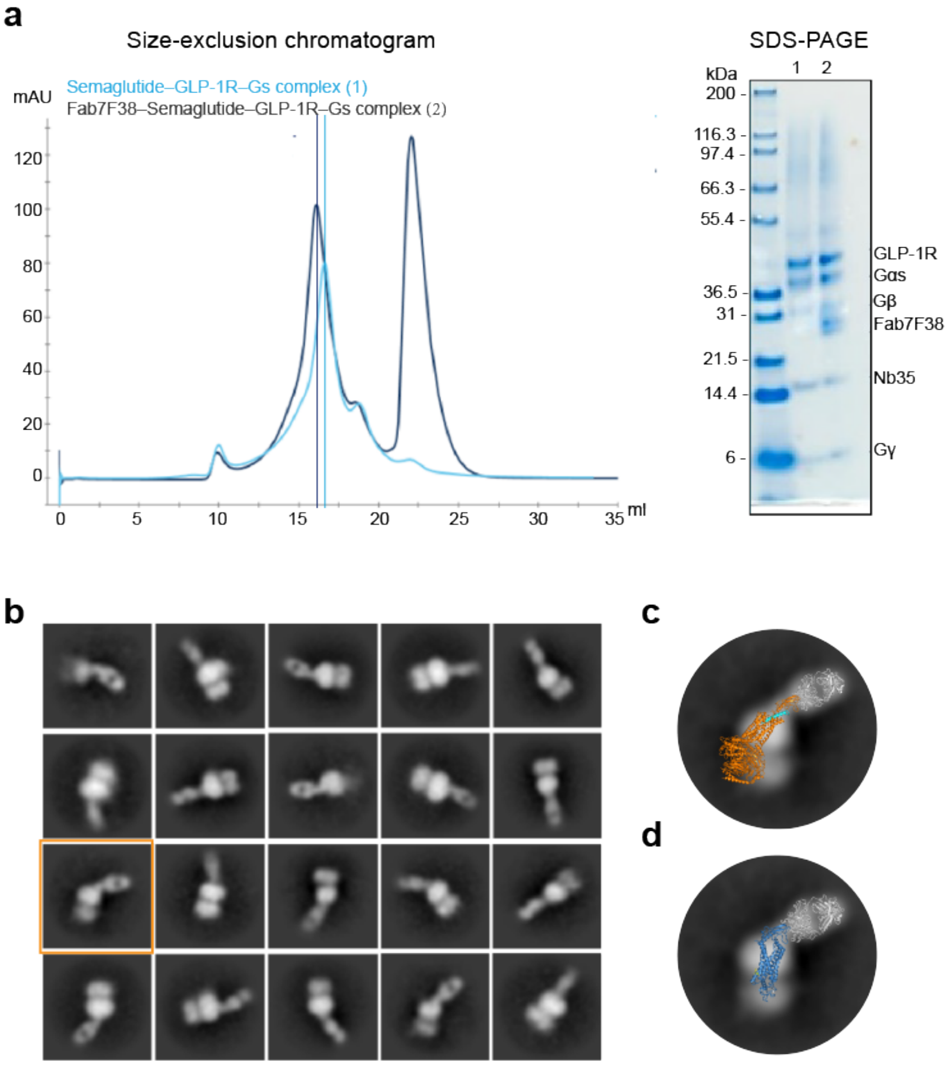

Supplementary Fig. 5. Negative staining EM data of the Fab7F38–semaglutide–GLP-1R–Gs–Nb35 complex.

a Size-exclusion chromatogram and SDS-PAGE of the semaglutide–GLP-1R–Gs–Nb35 complex with and without Fab7F38. **b** 2D class averages based on 9101 accepted particles. **c** Superposition of a model of the Fab7F38-bound GLP-1–GLP-1R–Gs complex on to 2D class average highlighted in b, illustrating the conformational discrepancies between the selected 2D class and the active-state model. Fab7F38 is shown in grey, GLP-1R–Gs in orange, and GLP-1 in cyan. **d** Fit of the inactive GLP-1R–Fab7F38 crystal structure in to the densities of the 2D class average highlighted in b. Fab7F38 and GLP-1R is depicted in grey and blue cartoon, respectively. The superpositions in c and d are guided by Fab7F38 and its density features. Source data is provided as a Source data file.

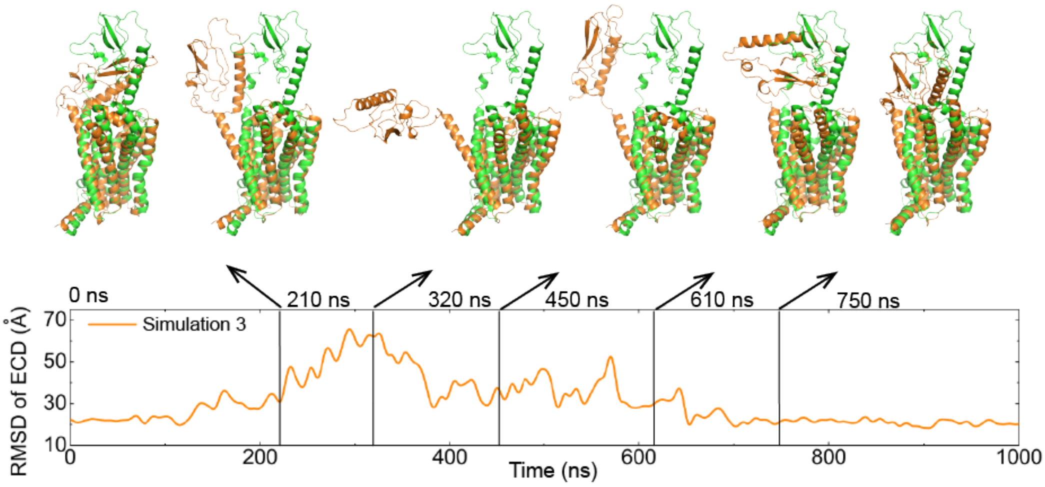

Supplementary Fig. 6. Comparison of the third trajectory snapshots with the active GLP-1R–GLP-1 structure. The full-length active GLP-1R structure (PDB: 5VAI) and the snapshots of trajectory 3 are shown as green and yellow cartoons, respectively. Main chain r.m.s.d. values of the ECD versus simulation time are shown for 1 μs MD simulations, values are calculated from snapshots at 100 ps intervals.
