# Supplementary material for: Full-length human GLP-1 receptor structure without orthosteric ligands
Source: Nat Commun. 2020 Mar 9;11:1272. doi: 10.1038/s41467-020-14934-5 (PMC7062719; doi:10.1038/s41467-020-14934-5)
Supplement: Supplementary file 7 — Description of Additional Supplementary Files [file 41467_2020_14934_MOESM7_ESM.pdf]

Title: Supplementary Movie 1.
Description: Molecular Dynamics (MD) simulation of the first trajectory (see Figure 5) reveals relatively stable mobility of the Extracellular Domain (ECD).

Title: Supplementary Movie 2.
Description: Molecular Dynamics (MD) simulation of the second trajectory (see Figure 5) reveals relatively stable mobility of the Extracellular Domain (ECD) similar to the first trajectory.

Title: Supplementary Movie 3.
Description: Molecular Dynamics (MD) simulation of the third trajectory (see Figure 5) reveals relatively large mobility of the Extracellular Domain (ECD).
